# Problem severity and waiting times for young people accessing mental health services

**DOI:** 10.1192/bjo.2020.103

**Published:** 2020-10-12

**Authors:** Julian Edbrooke-Childs, Jessica Deighton

**Affiliations:** Evidence Based Practice Unit, University College London & Anna Freud National Centre for Children and Families, Clinical, Educational and Health Psychology, UK; Evidence Based Practice Unit, University College London & Anna Freud National Centre for Children and Families, Clinical, Educational and Health Psychology, UK

**Keywords:** Child and adolescent, mental health, waiting time, access

## Abstract

**Background:**

Access to timely care is a quality standard underpinning many international healthcare models, and long waiting times for child and adolescent mental health services are often reported as a barrier to help-seeking.

**Aims:**

The aim of this study was to examine whether young people with more severe problems have shorter waiting times for mental health services.

**Method:**

Multilevel multinomial regression analysis controlling for service-area deprivation, age, gender, ethnicity, referral source and contextual factors was conducted on *N* = 21 419 episodes of care (mean age 12.37 years (s.d. = 3.71), 11 712 (55%) female) using data from child and adolescent mental health services.

**Results:**

There was high variation in waiting times, which ranged from 0 days to 1629 days (mean 50.65 days (s.d. = 78.03), median 32 days). Compared with young people with less severe problems young people with severe problems, self-harm, psychosis or eating disorders were less likely to experience longer waiting times. Moreover, referrals from sources other than primary care were generally less likely to have longer waiting times than referrals from primary care sources, especially referral from accident and emergency services.

**Conclusions:**

The findings suggest that young people with more severe problems had shorter waiting times. Intermediary information and resources for support before access to services is needed to prevent escalation of problems and to support individuals and families while waiting for care. Interventions to reduce waiting times should be considered without compromising on the quality and experience of care that young people and families deserve when seeking help.

## Background

Access to timely care is a quality standard underpinning many international healthcare models. It is especially important in the treatment of mental health problems as a longer duration without treatment may contribute to the development of more intractable problems.^1^ In England, child and adolescent mental health services are recommended to provide treatment within 2 weeks of referral for psychosis, between 2 and 4 weeks of referral for eating disorders, and within 18 weeks for all referrals.^2^ Recent government guidance for child and adolescent mental health services is testing a maximum waiting time of 4 weeks for routine cases and 1 week for urgent cases in some regions.^3,4^ It is therefore important to examine existing waiting times, how individuals are prioritised, and in particular whether young people with more severe problems have shorter waiting times.

Professionals report that waiting time is a common barrier to referring to child and adolescent mental health services and that increasing levels of demand and complexity make it ever more difficult to meet levels of need, resulting in long waiting times.^5^ There is anecdotal evidence that, because of limited resources and increased levels of demand, thresholds for accessing child and adolescent mental health services may have increased to prioritise those with most severe needs^6^ and empirical investigation of this is urgently needed.

## Waiting times and impact on help-seeking

According to the gateway provider model, help-seeking agents make decisions about accessing child and adolescent mental health services based on perceptions and knowledge of the structural and systemic characteristics of their environment and their predisposing factors, levels of need and enabling factors.^7^ Help-seeking agents, such as young people and parents or carers, often report dissatisfaction with long waiting times for mental health services and that this is a barrier to help-seeking and accessing care, which moreover may result in deterioration and risk of problems escalating while waiting for care^8^ (also see a study by Anderson et al^9^ for a scoping review).

Waiting times are important in relation to both initial access (for example, assessment) and start of treatment (for example, post-assessment). It is known that treatment waiting times may be particularly challenging for child and adolescent mental health services.^10^ Previous studies examining data from child and adolescent mental health services have found that longer waiting times were associated with lower levels of subsequent treatment engagement.^11^ A range of approaches have been examined to reduce waiting times, including guidance for the identification and management of mental health problems, the implementation of dedicated centres focused on assessment, and service re-design models (see a study by Ansell et al^12^ for a systematic review).

## Factors linked to waiting times

There is a high level of heterogeneity in waiting times reported in previous studies.^13–19^ A recent child and adolescent mental health survey in England found that 61% of those with a mental health problem accessed specialist services in less than 10 weeks, 18% in 10 weeks to 6 months, and 21% in more than 6 months.^20^ Examining waiting times is complex given the number of environmental and individual characteristics related to mental health, help-seeking behaviour and service provision. Waiting times may differ between services and therefore, analysis needs to account for the fact that data are nested within services. Waiting times may be affected by deprivation as areas of higher deprivation may have more young people with mental health problems and lower levels of service provision.

The pattern of findings from previous studies on the association between individual characteristics and waiting times is mixed. Some studies have found that males were more likely to have longer waiting times than females or that age was negatively associated with waiting times;^21–24^ however, other studies have found that age was positively associated with waiting times.^21^ Similarly, there is conflicting evidence about whether referrals from some settings such as education are associated with longer or shorter waiting times.^13,21^

Few studies have examined the association between severity and waiting times. Evidence suggests that externalising problems may be associated with longer waiting times than other types of problems^23^ (also see a study by Smith et al^25^) and that higher levels of severity are associated with shorter waiting times for attention-deficit and hyperactivity disorder (ADHD) diagnosis,^22^ suggesting that services may be able to appropriately triage cases with higher levels of severity. To the best of our knowledge, no previous study has examined whether young people with more severe problems have shorter waiting times for mental health services, controlling for service-level variation, deprivation, demographic characteristics, referral source and contextual factors.

## Aims

The aim of the present study was to examine whether young people with more severe problems have shorter waiting times for mental health services. We hypothesised that young people with more severe problems would be less likely to experience longer waiting times than young people with less severe problems.

## Method

### Participants and procedure

The data corpus was collected from child and adolescent mental health services, including those participating in a programme to implement evidence-based practice between 2011 and 2015.^26^ Episodes of care were included in the present analysis if young people were aged ≤25 years, had complete demographic characteristics (for example age, gender), had a referral date and a date of first contact, were referred to services between 2011 and 2015, and had complete information on problem severity (see Measures). This resulted in a final data-set of *N* = 21 419 episodes of care (mean age 12.37 (s.d. = 3.71) years, 11 712 or 55% female). It should be noted that in the data corpus, pseudonymised data are uploaded according to episodes of care; therefore, it is possible that a young person may have been included under more than one episode of care.

Detailed demographic characteristics are shown in Table 1.

**Table 1:** Descriptive statistics for study variables (*n* = 21 419)

Variable	*n*	%
Service-area deprivation		
IDACI band 0	6858	32
IDACI band 1	8750	41
IDACI band 2	1312	6
IDACI band 3	4499	21
*Demographics*		
Age		
0–12 years	8858	41
13–15 years	8055	38
≥16 years	4476	21
Gender, female	11 712	55
Ethnicity		
White British	13 505	63
White Other	779	4
Mixed-race	739	3
Asian	1343	6
Black	835	4
Other	632	3
Not stated	3586	17
Referral source^a^		
Primary care	9898	46
Self-referral	988	5
Social care/youth justice	807	4
Education	1675	8
Child health	1087	5
Accidence and emergency	998	5
Mental health	1742	8
Other	2153	10
Missing	2071	10
Contextual factors		
Home	6493	30
School	6394	30
Community	2538	12
Engagement	1008	5
Severity		
Self-management	6363	30
Severe problems	1931	9
Self-harm	1168	5
Psychosis	262	1
Eating disorder	403	2
Attention-deficit hyperactivity disorder	1254	6
Emotional	1950	9
Unclassified	3437	16
Other	4651	22
Waiting times^a^		
0–2-week wait	5644	26
3–4-week wait	4147	19
5–18-week wait	10 033	47
≥19-week wait	1595	7

IDACI, Income Deprivation Affecting Children Index.

a. Referral source and waiting times do not sum to 100% because of rounding.

### Ethical considerations

The present analysis involved secondary analysis of anonymised administrative data and therefore, ethical review was not required.^27^

### Measures

#### Deprivation

We matched data on services to the normalised Income Deprivation Affecting Children Index (IDACI) to generate an average score based on the lower layer super output area in each service's catchment area. Scores were then transformed into bands using the following established categories^28^: <0.2 (least deprived) band 0; 0.2–0.249 band 1; 0.25–0.299 band 2; and 0.3–0.4 (most deprived) band 3; there were no IDACI scores >0.4.

#### Demographic characteristics

Age, gender and ethnicity were recorded by services as part of routine data recording. For the main analysis, age was coded as 13–15 years and ≥16 years (where 0–12 years was selected as the reference category to facilitate interpretation). Ethnicity was captured using the categories from the 2001 census and was generally based on self-report by the parent/carer or the young person. These were grouped for analysis as follows:^29^ White British (as the ethnic majority group), White other (including Irish and other White background), mixed-race (including mixed White and Black Caribbean, mixed White and Black African, mixed White and Asian, and any other mixed background), Asian (including Indian, Pakistani, Bangladeshi, and other), Black or Black British (including Caribbean, African, and other), other ethnic groups (including Chinese and other), and not stated.

#### Referral source

As used in previous research, referral source was recorded by services using 36 indicators, which were grouped into eight study variables for the present analysis, which are shown in Table 1.^30^ In the main analysis, referral from primary care was selected as the reference category as it was the largest group.

#### Contextual factors

Contextual factors were identified using four items of the Current View questionnaire.^31^ Clinicians rated the extent to which young people were experiencing problems in four contextual areas: ‘Home’, ‘School, work or training’, ‘Community’, and ‘Service engagement’ (coded 1 for ‘moderate’ or ‘severe’ and 0 for ‘mild’ or not applicable).

#### Problem severity

Problem severity was identified using an algorithm^32,33^ based on 30 items of the clinician-rated Current View questionnaire.^31^ The algorithm categorises young people into 18 mutually exclusive needs-based groups, but as there were no young people in the ‘suggestive of borderline personality’ group, 17 subgroups were initially used. However, to minimise including underpowered groups in the main analysis, we used nine groups and categorised those occurring with a frequency of ≤5% as ‘other’ problems (see point (i) below); ‘psychosis’ and ‘eating disorder’ were retained as separate groups despite occurring with frequencies of ≤5% as they were of central importance to the aims of the present research as theory and policy suggest these groups may experience shorter waiting times (see Background). The nine groups used were as follows:

- ‘signposting and self-management advice’ referring to young people for whom clinicians rated a maximum of one problem as moderate;
- ‘difficulties of severe impact’ (for example young people for whom clinicians rated at least two problems as severe);
- ‘self-harm’;
- ‘psychosis’;
- ‘eating disorder’;
- ‘ADHD’;
- ‘emotional problems’;
- ‘difficulties not covered by other groupings’ or unclassified problems; and
- ‘other problems’ (i.e. ‘bipolar disorder’, ‘depression’, ‘generalised anxiety problems’, ‘behavioural and/or conduct disorder’, ‘obsessive–compulsive disorder’, ‘autism’, ‘co-occurring behavioural and emotional difficulties’, ‘post-traumatic stress disorder’ and ‘social anxiety disorder’).

#### Waiting time

Waiting time was computed as the difference between date of referral and date of first event or contact (for example initial assessment). To enable comparison with a recent national survey,^20^ waiting times were grouped into: less than 10 weeks (0–69 days), 10 weeks to 6 months (70–168 days), and more than 6 months (>168 days). Based on non-mandatory guidelines,^2^ and given the non-normal distribution of waiting times found in the present data and previous research (see Background), waiting times were grouped for the main analysis into: 0–2-week wait (0–14 days), 3–4-week wait (15–28 days), 5–18-week wait (29–126 days), and ≥19-week wait (≥127 days).

### Statistical analysis

To examine whether young people with more severe problems had shorter waiting times for mental health services, accounting for the nesting of episodes of care in services and controlling for deprivation, age, gender, ethnicity and contextual factors multilevel multinomial logistic regressions were conducted in Stata 14.^34^ Four preparatory models were estimated.

In model 0 (null model) the variance explained in waiting time at the service-level was examined and no predictors were added. The intraclass correlation coefficient was 25% indicating that there was significant service-level variation in waiting times and confirming that multilevel modelling was the appropriate statistical analysis. In model 1, we examined whether service-level deprivation explained variation in waiting time using IDACI bands, where band 0 representing the lowest level of deprivation was selected as the reference group to facilitate interpretation. In model 2, demographic characteristics were added: female; age coded 13–15 and ≥16 years with 0–12 years as the reference category; and ethnicity with the White British group as the reference category as it was the largest group. In model 3, referral source was added with primary care as the reference category and the four contextual factors were added, which were dummy coded as young people's contextual factors were not mutually exclusive. Problem severity was added to the final model using the nine problem groups, where the ‘Signposting and self-management advice’ group was selected as the reference category as it was the largest group, referring to young people for whom clinicians rated a maximum of one problem as moderate.

The likelihood ratio test was used to compare successive models, which were significant and all variables were therefore retained in the final model. In particular, the likelihood ratio test was significant for the final model compared to model 3: χ^2^(24) = 336.16, *P* < 0.001.

## Results

There was high variation in waiting times, which ranged from 0 days to 1629 days (mean 50.65 days (s.d. = 78.03), median 32 days). Overall, 16 737 (78%) young people waited less than 10 weeks, 3902 (18%) waited between 10 weeks and 6 months and 780 (4%) waited more than 6 months. The results of the final model (with problem severity in addition to service-level deprivation, demographic characteristics, referral source and contextual factors) are shown in Table 2.

**Table 2:** Multilevel multinomial regression analysis: service-area deprivation, demographics, referral source, contextual factors and severity predicting waiting times (*N* = 21 419)^a^

	3–4-week *v.* 0–2 week wait			5–18-week *v.* 0–2-week wait			≥19-week *v.* 0–2-week wait			
	OR	95% CI		OR	95% CI		OR	95% CI		
Service-area deprivation									
IDACI band 1 *v.* 0	0.89	0.47	1.71	1.08	0.56	2.06	1.78	0.92	3.44
IDACI band 2 *v.* 0	0.82	0.33	2.02	1.15	0.47	2.81	2.32	0.93	5.80
IDACI band 3 *v.* 0	0.68	0.28	1.68	0.95	0.39	2.33	0.72	0.29	1.79
Demographics										
Female *v.* male	0.95	0.86	1.04	**0**.**90**	0.83	0.97	**0**.**76**	0.67	0.86
13–15 *v.* 0–12 years	**0**.**64**	0.57	0.71	**0**.**52**	0.48	0.57	**0**.**52**	0.45	0.60
≥16 *v.* 0–12 years	**0**.**60**	0.53	0.68	**0**.**42**	0.38	0.47	**0**.**36**	0.30	0.43
White other *v.* White British	0.92	0.72	1.18	1.08	0.88	1.33	1.23	0.89	1.69
Mixed-race *v.* White British	0.98	0.77	1.25	1.11	0.90	1.37	1.00	0.71	1.41
Asian *v.* White British	**0**.**63**	0.50	0.78	0.92	0.76	1.10	**0**.**63**	0.45	0.87
Black *v.* White British	**0**.**55**	0.42	0.71	**0**.**77**	0.63	0.94	1.16	0.86	1.56
Other *v.* White British	**0**.**70**	0.53	0.94	0.91	0.72	1.15	**0**.**52**	0.33	0.82
Not stated *v.* White British	**1**.**34**	1.17	1.53	1.03	0.91	1.16	0.87	0.73	1.04
Referral source and contextual factors										
Self *v.* primary	**0**.**56**	0.45	0.69	**0**.**49**	0.41	0.58	0.88	0.68	1.15
Social care/youth justice *v.* primary	**0**.**50**	0.40	0.63	**0**.**41**	0.34	0.49	**0**.**38**	0.28	0.53
Education *v.* primary	**0**.**83**	0.70	0.98	**0**.**61**	0.52	0.70	**0**.**40**	0.31	0.52
Child health *v.* primary	0.83	0.66	1.03	0.86	0.71	1.03	0.99	0.76	1.29
Accident and Emergency *v.* primary	**0**.**14**	0.12	0.18	**0**.**10**	0.08	0.12	**0**.**12**	0.08	0.17
Mental health *v.* primary	**0**.**41**	0.35	0.49	**0**.**42**	0.37	0.49	**0**.**82**	0.68	1.00
Other *v.* primary	**0**.**67**	0.57	0.79	**0**.**68**	0.59	0.78	**0**.**48**	0.38	0.60
Missing *v.* primary	**0**.**70**	0.58	0.84	**0**.**35**	0.30	0.42	**0**.**17**	0.12	0.23
Home	0.93	0.84	1.04	0.96	0.88	1.05	1.07	0.92	1.24
School	1.02	0.91	1.14	0.94	0.86	1.03	1.00	0.86	1.16
Community	1.03	0.88	1.20	1.10	0.97	1.26	0.83	0.67	1.03
Engagement	0.83	0.68	1.03	**0**.**78**	0.65	0.93	1.09	0.82	1.46
Severity										
Severe problems *v.* self-management	**0**.**79**	0.67	0.94	**0**.**73**	0.63	0.84	**0**.**74**	0.59	0.94
Self-harm *v.* self-management	**0**.**48**	0.40	0.58	**0**.**35**	0.29	0.41	**0**.**29**	0.21	0.42
Psychosis *v.* self-management	**0**.**53**	0.37	0.78	**0**.**39**	0.28	0.54	**0**.**29**	0.14	0.61
Eating disorder *v.* self-management	1.02	0.78	1.35	**0**.**51**	0.39	0.67	**0**.**46**	0.26	0.80
ADHD *v.* self-management	**1**.**32**	1.05	1.67	**1**.**66**	1.36	2.03	**1**.**76**	1.35	2.31
Emotional problems *v.* self-management	1.13	0.95	1.34	**1**.**18**	1.02	1.37	1.07	0.85	1.35
Unclassified problems *v.* self-management	0.95	0.82	1.09	0.97	0.86	1.10	0.91	0.76	1.10
Other *v.* self-management	0.93	0.82	1.06	1.01	0.90	1.12	0.87	0.73	1.03

IDACI, Income Deprivation Affecting Children Index; ADHD, attention-deficit hyperactivity disorder.

a. Odds ratios in bold are significant at least at the *P* < 0.05 level.

Compared with boys, girls were less likely to wait 5–18 weeks and ≥19 weeks than 0–2 weeks. Compared with young people aged 0–12 years, young people aged 13–15 years or ≥16 years were less likely to wait 3–4 weeks, 5–18 weeks and ≥19 weeks than 0–2 weeks. Compared with White British young people, Asian young people were less likely to wait 3–4 weeks and ≥19 weeks than 0–2 weeks. Compared with White British young people, Black young people were less likely to wait 3–4 weeks and 5–18 weeks than 0–2 weeks. Compared to White British young people, young people from ‘other’ ethnic backgrounds were less likely to wait 3–4 weeks and ≥19 weeks than 0–2 weeks. Compared with White British young people, young people with not stated ethnic backgrounds were more likely to wait 3–4 weeks than 0–2 weeks.

Referrals from sources other than primary care were consistently less likely to have longer waiting times than referrals from primary care sources, except for referrals from child health (and self-referrals when comparing ≥19-week wait to 0–2-week wait) which were not significantly different. In particular, compared with referrals from primary care sources, referrals from accident and emergency services were less likely to wait 3–4 weeks, 5–18 weeks, and ≥19 weeks compared with 0–2 weeks. Compared with young people without contextual problems in service engagement, young people with contextual problems in service engagement were less likely to wait 5–18 weeks than 0–2 weeks.

The hypothesis that young people with more severe problems would have shorter waiting times for mental health services than young people with less severe problems was supported. Compared with young people in the signposting and self-management advice group, young people with severe problems, self-harm, and psychosis were less likely to wait 3–4 weeks, 5–18 weeks and ≥19 weeks than 0–2 weeks. In addition, compared with young people in the signposting and self-management advice group, young people with eating disorders were less likely to wait 5–18 weeks and ≥19 weeks than 0–2 weeks. Finally, compared with young people in the signposting and self-management advice group, young people in the ADHD group were more likely to wait 3–4 weeks, 5–18 weeks, and ≥19 weeks than 0–2 weeks, and young people in the emotional problems group were more likely to wait 5–18 weeks than 0–2 weeks.

## Discussion

The aim of the present study was to examine whether young people with more severe problems had shorter waiting times for mental health services, using multilevel multinomial regression analysis controlling for service-area deprivation, age, gender, ethnicity, referral source and contextual factors. We hypothesised that young people with more severe problems would be less likely to have longer waiting times than young people with less severe problems.

### Main findings and comparison with findings from other studies

In line with previous studies, there was a high level of heterogeneity in waiting times.^13–19^ We found shorter waiting times than reported in a recent child and adolescent mental health survey – in the present study, 16 737 (78%) young people waited less than 10 weeks, 3902 (18%) waited between 10 weeks and 6 months, and 780 (4%) waited more than 6 months – although it should be noted that different methodologies including operationalisations of waiting times were used.^20^ The hypothesis that young people with more severe problems would have shorter waiting times for mental health services than young people with less severe problems was supported.

Compared with young people in the signposting and self-management advice group, where clinicians rated a maximum of one problem as moderate, young people with severe problems, self-harm and psychosis were less likely to have longer waiting times. In addition, compared with young people in the signposting and self-management advice group, young people with eating disorders were generally less likely to have longer waiting times.

Finally, compared with young people in the signposting and self-management advice group, young people in the ADHD and emotional problems groups were more likely to have longer waiting times.

The findings of the present research are in line with previous studies suggesting that externalising problems may be associated with longer waiting times than other types of problems^23^ (also see a study by Smith et al^25^) and that higher levels of severity are associated with short waiting times for ADHD diagnosis when examining only those with ADHD^22^ (although in comparison with the self-management group in the present study, young people with ADHD were more likely to have longer waiting times).^22^ Moreover, the findings that young people with psychosis were less likely to wait 3–4 weeks, 5–18 weeks, and ≥19 weeks, and that young people with eating disorders were less likely to wait 5–18 weeks and ≥19 weeks, are in line with recommendations for child and adolescent mental health services in England to provide treatment within 2 weeks of referral for psychosis and between 2 and 4 weeks of referral for eating disorders.^2^

### Source of referrals

Referrals from sources other than primary care were consistently less likely to have longer waiting times than referrals from primary care sources, with some exceptions (see Results). In particular, compared with referrals from primary care sources, referrals from accident and emergency services were less likely to wait 3–4 weeks, 5–18 weeks, and ≥19 weeks compared with 0–2 weeks. These findings suggest a pattern of crisis responsiveness, in line with the findings that young people with more severe problems had shorter waiting times for mental health services.

### Waiting times and ethnicity

In addition, in some instances, young people from minority ethnic groups were less likely to have longer waiting times, which is in line with evidence that young people from minority ethnic groups are more likely to access mental health services through routes that are less likely to be voluntary.^30^

### Limitations

The present research addresses an important gap in the literature on whether young people with more severe problems have shorter waiting times for mental health services, controlling for service-level variation, deprivation, demographic characteristics, referral source and contextual factors. Nevertheless, limitations should be considered when interpreting the findings of the present research. The data were routinely collected from child and adolescent mental health services and were collected from one country. In particular, the aim of the present study was to examine whether young people with more severe problems have shorter waiting times for mental health services. In the present research, it was not possible to examine whether waiting times differed pre- or post-assessment, and one reason for the lower waiting times found in the present research may be that they do not represent post-assessment waiting times, which may be particularly challenging for child and adolescent mental health services.^10^

In addition, findings may not generalise to other countries, especially with different policies and targets on waiting times and referral routes to child and adolescent mental health services. We controlled for a number of factors based on past theory and research (see Background); however, it is possible that other environmental and individual characteristics may explain the pattern of findings and are associated with severity and waiting times.

### Further research

Importantly, in the present research we examined problem severity based on clinician ratings, and it is crucial to review whether the findings of the present research are in line with the views and lived experiences of help-seeking agents, particularly young people and parents or carers. One research question of particular interest is whether young people's self-reported levels of need, risk and distress at time of referral are associated with differential waiting times. Moreover, interventions to reduce waiting times may result in worse treatment outcomes and experiences if they are not evidence-based and informed by clinical and lived expertise. A multifaceted approach to reducing waiting times is needed that additionally accounts for improving treatment outcomes and experiences.

### Implications

The findings of the present research and the extant literature suggest that young people with more severe problems have shorter waiting times compared with young people with less severe problems. The findings of the present research build on anecdotal evidence that thresholds for accessing child and adolescent mental health services may have increased to prioritise those with most severe needs.^6^ Intermediary information and resources for support before access to services is needed to prevent escalation of problems and to support individuals and families while waiting for care, especially considering that individuals and families may be experiencing problems that are subjectively far from ‘less severe’.

Information and resources could include online-supported self-management, access to voluntary or third-sector organisations, or signposting to support not accompanied by a professional (for example community, peer or family support).^35^ In addition, complimentary pathways for the early identification of difficulties at an early stage of development or presentation are needed, as findings from the present research suggest that these young people may be more likely to have longer waiting times for mental health services. Although it is clearly important to prioritise those with high need, it is also important to not miss opportunities for early intervention for those with emerging difficulties, as this may be when the biggest impact might be seen from intervention.^1^ Primary prevention at the levels of universal, targeted to disproportionally affected groups, and those experiencing emerging difficulties, in addition to mental health promotion that focuses on empowering all young people with the resources to actively manage their mental health, are crucial parts of this continuum of care. Any interventions and approaches to reducing waiting times should be considered without compromising on the quality and experience of care that young people and families deserve when seeking help.

**Fig. 1:**
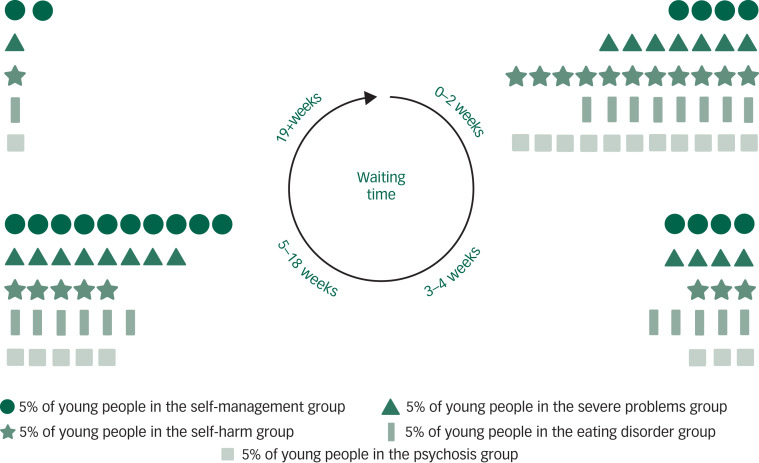
Young people and waiting times for mental health services: Summary of key findings for the main problem severity groups. In the self-management group, approximately 20% of young people waited 0–2 weeks, 20% waited 3–4 weeks, 50% waited 5–18 weeks and 10% waited 19+ weeks. In the severe problems group, approximately 35% of young people waited 0–2 weeks, 20% waited 3–4 weeks, 40% waited 5–18 weeks and 5% waited 19+ weeks. In the self-harm group, approximately 55% of young people waited 0–2 weeks, 15% waited 3–4 weeks, 25% waited 5–18 weeks and 5% waited 19+ weeks. In the eating disorder group, approximately 40% of young people waited 0–2 weeks, 25% waited 3–4 weeks, 30% waited 5–18 weeks and 5% waited 19+ weeks. In the psychosis group, approximately 55% of young people waited 0–2 weeks, 15% waited 3–4 weeks, 25% waited 5–18 weeks and 5% waited 19+ weeks.

## Data Availability

The data that support the findings of this study are available on request from the corresponding author, J.E.-C. The data are not publicly available due to license for use for the present study from the Child Outcomes Research Consortium (CORC).
